# The effects of feedback timing and frequency on the acquisition of cardiopulmonary resuscitation skills of health sciences undergraduate students: A 2 x 2 factorial quasi randomized study

**DOI:** 10.1371/journal.pone.0220004

**Published:** 2019-07-17

**Authors:** Kazunori Akizuki, Ryohei Yamamoto, Kazuto Yamaguchi, Jun Yabuki, Yukari Ohashi

**Affiliations:** 1 Department of Physical Therapy, Kobe International University, Kobe, Hyogo, Japan; 2 Department of Rehabilitation, Kyushu University of Nursing and Social Welfare, Tamana, Kumamoto, Japan; 3 Misato Central General Hospital, Misato, Saitama, Japan; 4 Ibaraki Prefectural University of Health Sciences Hospital, Ami, Ibaraki, Japan; 5 Department of Physical Therapy, Ibaraki Prefectural University of Health Sciences, Ami, Ibaraki, Japan; University of Palermo, ITALY

## Abstract

**Background:**

High-quality training is required to improve the cardiopulmonary resuscitation (CPR) skills. Although it has been reported that the use of a feedback device is effective, the effects of feedback timing and frequency on CPR training have not been investigated. The aim of this study was to clarify the influence of feedback frequency and timing on the acquisition of CPR skills.

**Methods:**

Sixty-eight undergraduates were first divided into female (n = 32) and male (n = 36) groups, and randomly assigned to one of four groups for each sex: concurrent-100%, concurrent-50%, terminal-100%, and terminal-50% feedback groups. The randomization was performed using a lottery method. This study consisted of a pre-test, practice sessions, a post-test, and a follow-up test. In the practice sessions, the participants performed six 2-minute CPR sessions in accordance with the condition assigned using mannequins and feedback devices. The post-test was conducted 24 hours after the completion of the practice sessions and the follow-up test was conducted 3 months after the completion of the practice sessions. The primary outcome of the study was the overall score at the follow-up test.

**Results:**

The results of the overall score at the follow-up test for each group were 88.2 ± 9.6% for concurrent-100%, 92.2 ± 6.4% for concurrent-50%, 82.6 ± 16.4% for terminal-100%, and 85.2 ± 16.9% for terminal-50%. We did not find any statistically significant difference for the overall score at the follow-up test among the four groups (p = 0.173). The ANOVA for the test sessions revealed that there were no significant main effects of feedback timing (p = 0.135) or frequency (p = 0.765), and no significant interaction between timing and frequency (p = 0.997).

**Conclusion:**

The present study reveals that the use of feedback devices is an important factor for higher quality CPR training, regardless of the timing and frequency with which they are used.

## Introduction

Out-of-hospital cardiac arrest (OHCA) is a major global public health issue and one of the leading causes of death in many regions worldwide [1–3]. Cardiopulmonary resuscitation (CPR) and the use of public-access automated external defibrillators (AEDs) by bystanders has been shown to increase the survival rate after OHCA [4,5]. However, it has been reported over the years that the proportion of bystander CPR performed and the quality of CPR remains low [6]. Evidence highlighting the importance of the quality of CPR has been accumulated. It has also been clarified that high-quality CPR improves the survival rates after OHCA and improves patients’ outcomes [7–10]. As a standard for high-quality CPR, international guidelines recommend a chest compression depth of 50–60 mm, a compression rate of 100–120 compressions/min, a compression fraction > 60%, and a ventilation rate of 6/min [11].

High-quality training is required to improve the CPR quality. Recently, some randomized controlled trials (RCTs) reported that training using feedback devices is effective for improving CPR skills [12–14]. These RCTs clarified that providing real-time visual feedback has a greater learning effect than the conventional instructor-based feedback. Thus, there is high-grade evidence to suggest that the use of the feedback device should be the standard for CPR skill acquisition [15].

Although the effectiveness of using a feedback device and the influence of the time for which it is used have been clarified by previous research studies [12–14], to our knowledge, the effects of feedback timing and frequency on the acquisition of CPR skills have not been investigated. In the field of motor learning research, it has been reported that the timing and frequency of feedback affects the results of motor learning [16–19]. In the current CPR training, the timing and frequency of feedback during training does not receive much attention; instructors provide feedback concurrently (i.e., during task execution) and frequently during CPR training without being particularly conscious of the effects of their feedback delivery. It is not always advantageous for the long-term retention of acquired skills to provide frequent feedback or to provide feedback during task execution. Therefore, it is necessary to clarify the optimum feedback timing and frequency for CPR skill acquisition in order to improve the quality of CPR training. In this study, we aimed to clarify the influence of feedback frequency and timing on the acquisition of CPR skills.

## Materials and methods

### Study design and participants

This study received approval from the Mejiro University Ethics Review Committee (approval number: 15–008). Prior to initiation of the study, we explained the details regarding the experiment to all potential participants. Subsequently, we obtained written informed consent from all participants.

We conducted a prospective, 2 (feedback timing: concurrent versus terminal) × 2 (feedback frequency: 100% versus 50%) factorial, quasi-randomized study at Mejiro University located in Saitama-city, Japan. Undergraduate students were recruited voluntarily from the faculty of health sciences at Mejiro University between January 2016 and April 2016. Participants were included if they: had taken a Basic life support (BLS) course based on the Japan Resuscitation Council (JRC) Resuscitation Guidelines 2010 when they were a 1^st^ year student and were without physical or mental disabilities that would impact their CPR training. We excluded undergraduates who had taken a BLS course within a year prior to the initiation of this study, who had ever received BLS training based on the JRC Resuscitation Guidelines 2015, or who had ever received training using the feedback device used in this study.

### Equipment and devices

We used mannequins for BLS training (Resusci Anne^　^QCPR, Laerdal Medical, Stavanger, Norway) and feedback devices (SimPad SkillReporter, Laerdal Medical, Stavanger, Norway). The mannequins and the feedback devices were able to measure all the relevant aspects of CPR, including the compression depth, compression rate, and ventilation volume. These data were displayed graphically on the monitor of the feedback device in real time and were recorded in the device. Additionally, it also calculated the overall score, which reflects the comprehensive performance on CPR; the overall compression score, which is an index of comprehensive performance on chest compressions; and the overall ventilation score, which is an index of comprehensive performance on ventilation. The algorithm to calculate the overall score was developed by Laerdal Medical (Stavanger, Norway). These scores range from 0% to 100%, and when CPR performance deviates from the correct performance, the scores are reduced along a sinusoid curve. A larger deviation corresponds to a lowering of the score.

### Study procedures

**Fig 1:**
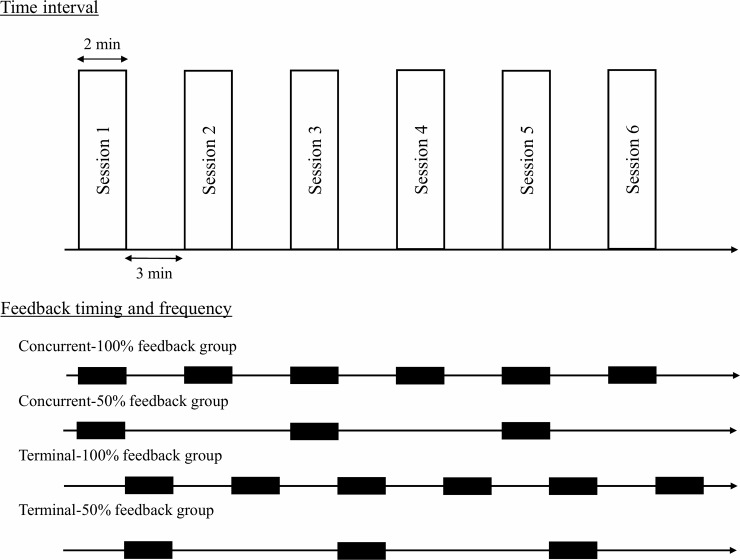
The time interval during the practice session and the timing and frequency at which each participant assigned to the four groups received feedback. The thick lines represent the period that is provided feedback from a device. The concurrent feedback groups were provided feedback during each session. The terminal groups were provided feedback immediately after completing each session. In the 50% feedback conditions, half the participants were provided with feedback for odd numbered sessions (session 1, 3, and 5), while the remaining half of the participants were provided with feedback for even numbered sessions (session 2, 4, and 6).

We first divided the participants into female and male groups to avoid bias in the number of participants per group and the female to male ratio. Next, we randomly assigned participants to one of four groups for each sex: concurrent-100%, concurrent-50%, terminal-100%, and terminal-50% feedback groups. The randomization was performed using a lottery method in blocks of 4 and 8. Concurrent or terminal represents the timing with which feedback was provided; for the concurrent feedback condition, feedback was provided during the session in real-time, whereas, for the terminal feedback condition, feedback was provided immediately after completing the session. The proportions represent the frequency with which feedback was provided; for the 100% feedback condition, feedback was provided for every session, whereas, for the 50% feedback condition, feedback was provided for half of the sessions (Fig 1).

Guidance for participants was carried out prior to measurement. In this guidance, we explained the main changes from the JRC Resuscitation Guidelines 2010 to the JRC Resuscitation Guidelines 2015 [20], how to implement CPR, and the content displayed on the feedback device and its interpretation. As the change to the guidelines, we informed all of the participants that the depth of chest compressions should be greater than 50 mm and less than 60 mm and that the rate of chest compressions should be greater than 100/min but within 120/min. For the explanation of how to implement CPR, we used a DVD prepared by the American Heart Association (AHA) [21]. Among all the contents recorded, only several parts of the DVD that related to this study (the chapters on BLS to adults, ventilation, and 1-Rescuer Adult BLS) were viewed by participants.

**Fig 2:**
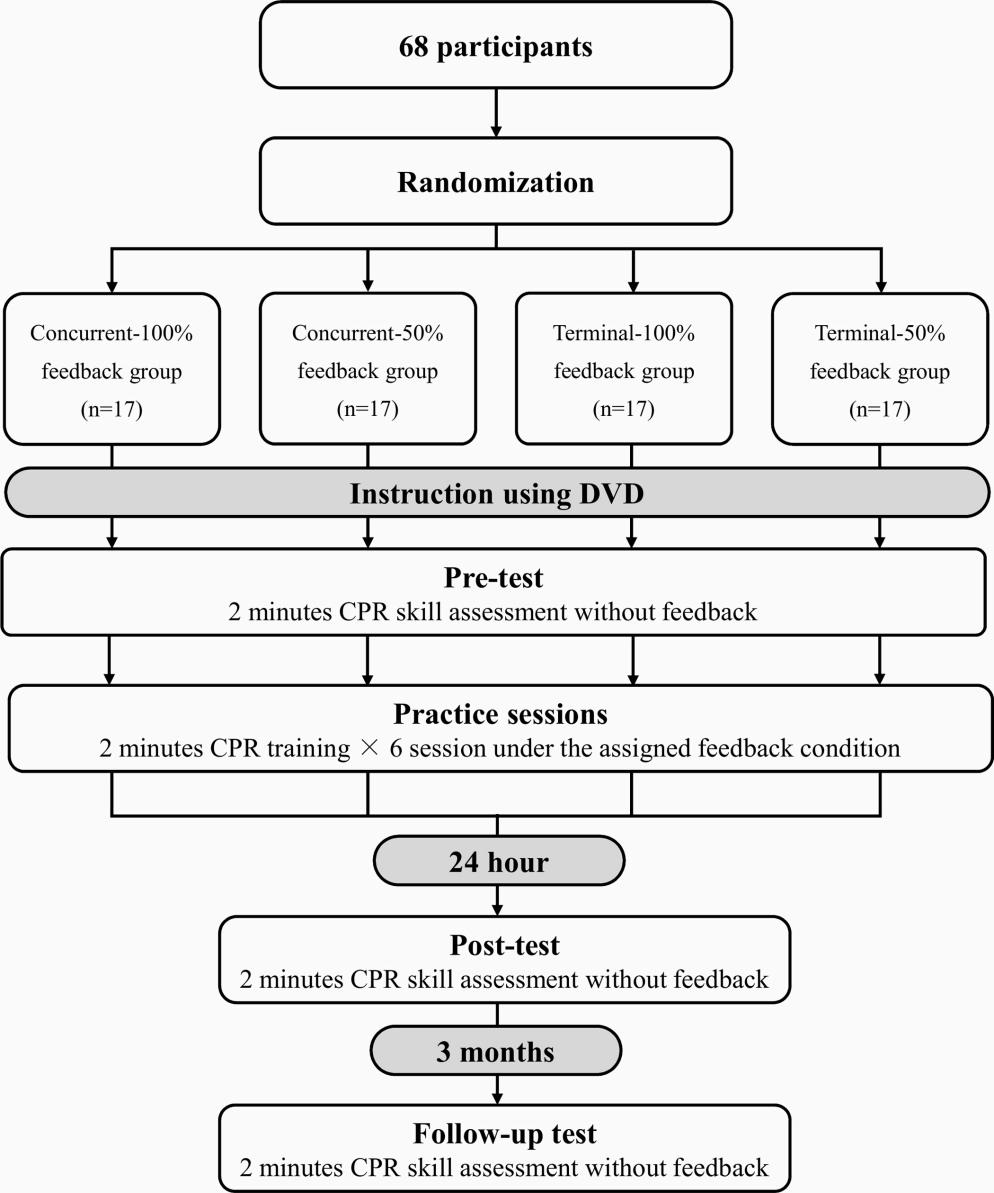
Experimental design to confirm the influence of feedback timing and feedback frequency on acquisition of CPR skills. To confirm the participant's skill levels before practice in the pre-test, all the participants conducted 2-minutes of CPR without the receipt of feedback. In the practice sessions, the participants performed six CPR sessions for 2 minutes in accordance with the condition assigned. The post-tests and follow-up tests were conducted with the same contents as the pre-test 24 hours and 3 months after completion of the practice sessions to determine the effects of feedback on retaining the acquired skills.

Measurements were conducted after the guidance was provided to all participants. There were pre-test, practice sessions, post-test, and follow-up test in this experimental design (Fig 2). To confirm the participant's skill level before practice in the pre-test, all the participants conducted 2-minutes of CPR without the receipt of feedback. In the practice sessions, the participants performed six CPRs for 2 minutes in accordance with the condition assigned. Participants assigned to the concurrent group were instructed to watch the monitor during CPR performance. Participants assigned to the terminal feedback group were instructed to browse the results displayed on the monitor of the feedback device for 2 minutes. Except for the period during which the participants were provided feedback, we ensured that the monitor of the feedback device could not be seen by participants. Three-minute breaks were inserted between sessions. The post-test was conducted with the same contents as the pre-test 24 hours after the completion of the practice sessions to determine the effects of feedback on the short-term retention of acquired skills. To discern the effects of feedback on the long-term retention of acquired skills, a follow-up test was conducted with the same contents as the pre-test 3 months after completion of the practice sessions. In all practice sessions and tests, participants used a facemask when they performed ventilation.

### Outcomes and data

The primary outcome of this study was the overall score at the follow-up test, which was conducted 3 months after the completion of the practice sessions under similar conditions among all groups. The secondary outcomes were changes in overall scores from pre-test to post-test, from pre-test to follow-up test, and from post-test to follow-up test; overall score in the practice session; overall compression score; and overall ventilation score. The other outcomes were the percentage of compressions performed with correct depth, percentage of compressions performed with correct rate, and percentage of ventilations performed with correct volume. The percentages of compressions performed with correct depth and rate were calculated based on the adequate range shown in the JRC Resuscitation Guidelines 2015 [20]. In the guidelines, it is specified that ventilation should ideally take approximately 1 second with a ventilation volume equal to the volume by which the victim's chest rises. However, concrete numerical values are not shown as to what ventilation volume is appropriate. Therefore, in this study, 400–700 ml was set as the appropriate range for the degree of ascertaining the rise of the manikin’s chest. This set value was decided by confirming the amount of ventilation in which the chest of the mannequin rises prior to the initiation of this study.

### Statistical analysis

To evaluate differences in the primary outcome among the groups, we performed one-way analysis of variance (ANOVA). Subsequently, to clarify the effect of feedback timing, feedback frequency, the measurement sequences for each measured outcome in the tests and practice sessions, and the interaction among these factors, we performed three-way analysis of variance (ANOVA). For test sessions, the overall score, the overall compression score, and the overall ventilation score were analyzed using 2 (feedback timing) × 2 (feedback frequency) × 3 (measurement sequence: pre, post, follow-up) ANOVA with repeated measures for the last factor. For practice sessions, the overall score, the overall compression score, and the overall ventilation score were analyzed using 2 (feedback timing: concurrent versus terminal) × 2 (feedback frequency: 100% versus 50%) × 6 (measurement sequence: session 1 to session 6) ANOVA with repeated measures for the last factor. If significant main effects or interactions were observed, two-way ANOVA or one-way ANOVA was performed as appropriate, and Tukey's multiple comparison test or independent t-test was performed depending on the results of ANOVA. Continuous variables were presented as mean ± standard deviation when normally distributed or as median and interquartile ranges if not.

Statistical analyses were performed using SPSS ver. 24 software (IBM Corp., Armonk, NY, USA), and the significance level was set at 5% (p-values < 0.05).

## Results

A total of 68 undergraduates (32 females, 36 males; mean age 21.0 ± 0.70 years; 2.0 ± 0.77 years since the last training) were included in this study. None of the participants had ever received BLS training based on JRC Resuscitation Guidelines 2015 or training using the feedback device used in this study.

### Overall score

**Fig 3:**
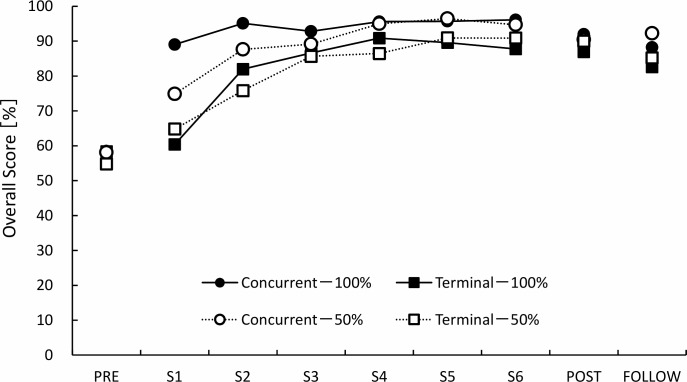
The transition of the overall score for each group. The overall score reflects the comprehensive performance on CPR including chest compression and ventilation. PRE: pre-test, S1: session 1, S2: session 2, S3: session 3, S4: session 4, S5: session 5, S6: session 6, POST: post-test, FOLLOW: follow-up test.

The results of the overall score at the follow-up test for each group were 88.2 ± 9.6% for concurrent-100%, 92.2 ± 6.4% for concurrent-50%, 82.6 ± 16.4% for terminal-100%, and 85.2 ± 16.9% for terminal-50%. We did not find any statistically significant difference for the overall score at the follow-up test among the four groups (p = 0.173). The results of the three-way ANOVA on the test sessions indicated that there were no significant main effects or interactions except for the main effect of the measurement sequence (p < 0.001) (See S2 Table). As only a significant main effect of the measurement sequence was observed, we performed Tukey’s multiple comparison test. The results indicated that significant differences were observed between the pre-test and post-test (mean difference: 32.4%, 95% confidence interval [CI]: 25.3–39.5, p < 0.001), and between the pre-test and follow-up test (mean difference: 29.6%, 95% CI: 22.6–36.7, p < 0.001), but there were no significant differences between the post-test and follow-up test (mean difference: 2.8%, 95% CI: -9.8–4.3, p = 1.00) (Fig 3).

In the practice session, the results of the three-way ANOVA indicated a significant main effect of feedback timing (p < 0.001) and the measurement sequences (p < 0.001) and a significant interaction between feedback timing and measurement sequences (p = 0.005). The post hoc analyses revealed that the concurrent condition was significantly superior to the terminal condition on sessions 1, 2, 4, 5, and 6 (p < 0.001, p < 0.001 p = 0.010, p < 0.001, p = 0.036, respectively).

### Overall compression score

**Fig 4:**
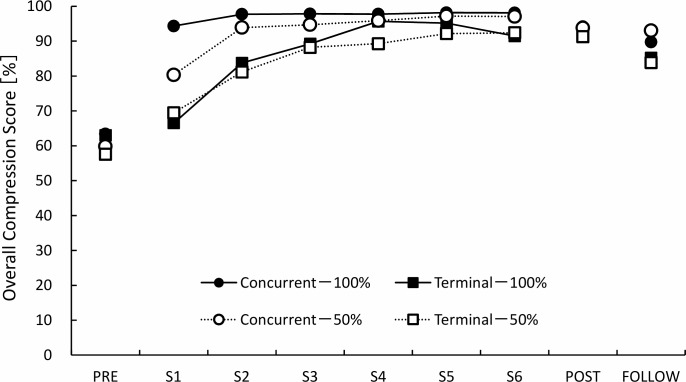
The transition of the overall compression score for each group. The overall compression score is an index of comprehensive performance on chest compressions. PRE: pre-test, S1: session 1, S2: session 2, S3: session 3, S4: session 4, S5: session 5, S6: session 6, POST: post-test, FOLLOW: follow-up test.

In the test sessions, the results of the three-way ANOVA indicated there were no significant main effects or interactions except for the main effect of the measurement sequence (p < 0.001). As only a significant main effect of the measurement sequence was observed, we performed Tukey’s multiple comparison test as a post hoc analysis. The results indicated that significant differences were observed between the pre-test and post-test (mean difference: 32.1%, 95% CI: 22.7–41.5, p < 0.001), and between the pre-test and follow-up test (mean difference: 27.0%, 95% CI: 17.6–36.4, p < 0.001), but there were no significant differences between the post-test and follow-up test (mean difference: -5.1%, 95% CI: -14.5–4.3, p = 0.581) (Fig 4).

In the practice session, the result of the three-way ANOVA indicated a significant main effect of feedback timing (p < 0.001), feedback frequency (p = 0.035), and the measurement sequences (p < 0.001), and a significant interaction between feedback timing and measurement sequences (p = 0.004). The 100% feedback condition (mean: 92.0%, 95% CI: 89.8–94.1) was superior to the 50% feedback condition (mean: 89.2%, 95% CI: 86.9–91.4). Additionally, the post hoc analyses revealed that the concurrent condition was significantly superior to the terminal condition on sessions 1, 2, 3, 4, and 5 (p = 0.001, p < 0.001 p = 0.005, p = 0.037, p = 0.003, respectively).

### Overall ventilation score

**Fig 5:**
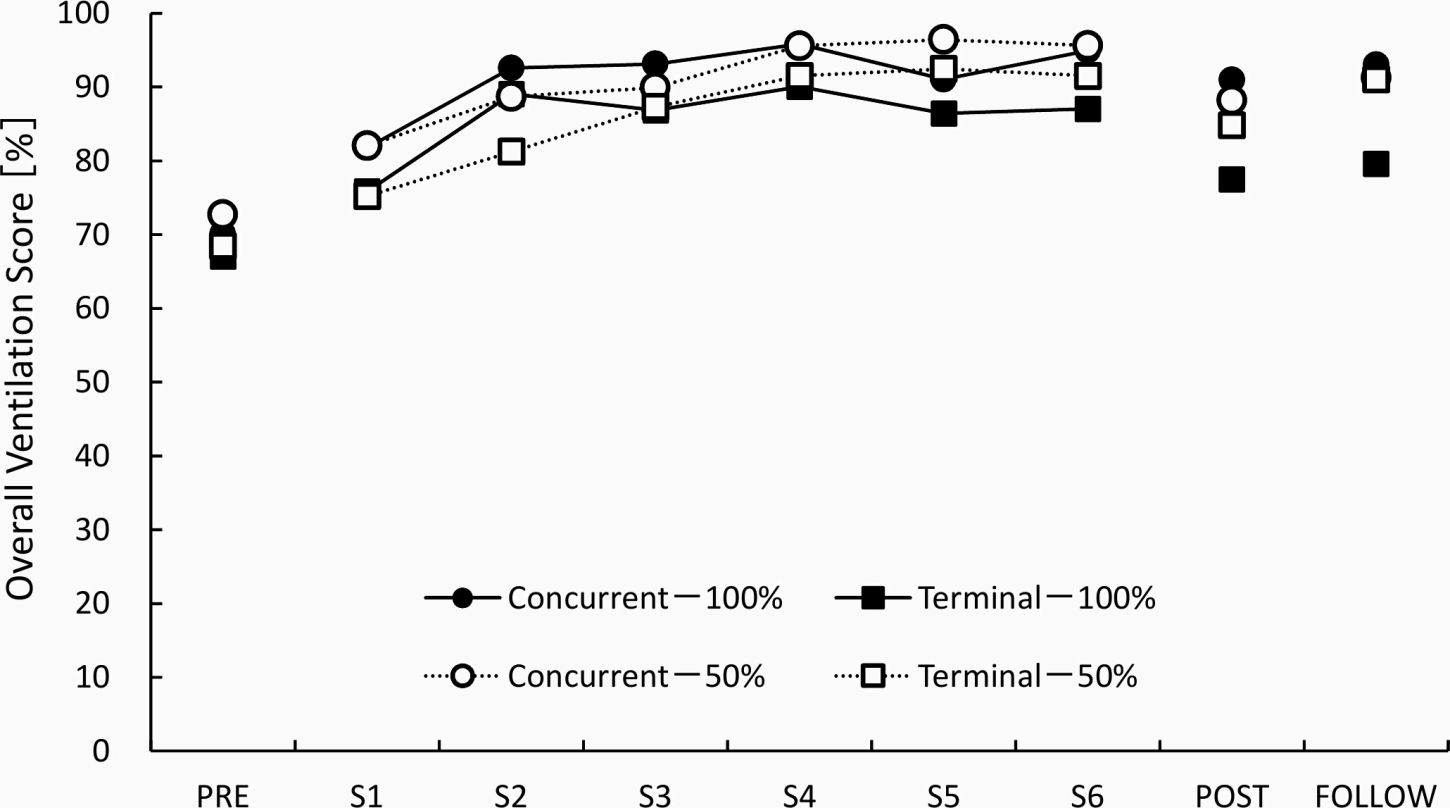
The transition of the overall ventilation score for each group. The overall ventilation score is an index of comprehensive performance on ventilation. PRE: pre-test, S1: session 1, S2: session 2, S3: session 3, S4: session 4, S5: session 5, S6: session 6, POST: post-test, FOLLOW: follow-up test.

In the test sessions, the results of the three-way ANOVA indicated there were no significant main effects or interactions except for the main effect of the measurement sequence. As only a significant main effect of the measurement sequence was observed, we performed Tukey’s multiple comparison test as a post hoc analysis. The results indicated that significant differences were observed between the pre-test and post-test (mean difference: 15.8%, 95% CI: 6.1–25.5, p < 0.001), and between the pre-test and follow-up test (mean difference: 19.2%, 95% CI: 9.5–28.9, p < 0.001), but there were no significant differences between the post-test and follow-up test (mean difference: 3.4%, 95% CI: -6.3–13.0, p = 1.00) (Fig 5).

In the practice session, the result of the three-way ANOVA showed a significant main effect of feedback timing (p = 0.002) and measurement sequences (p < 0.001). In the feedback timing, the concurrent condition (mean: 91.5%, 95%CI: 89.3–93.6) was superior to the terminal condition (mean: 85.9%, 95%CI: 83.2–88.7) (p = 0.002).

## Discussion

Previous studies have shown that using a feedback device can improve CPR performance during training [12–14]. However, the optimal timing and frequency of feedback is unknown. Therefore, we investigated the impact of feedback timing and feedback frequency on CPR skills for health sciences undergraduate students.

In this study, the training using the feedback device improved the overall score and the chest compression related indices by approximately 30% from pre-test to follow-up test. Ventilation related indicators showed less improvement in performance than chest compression indices. This superior learning effect is likely due to the use of a feedback device and it indicates the high quality of practice under an environment where the participants can obtain accurate feedback from the device. Other studies comparing the subjective instructor assessment and measured values by simulation manikins have revealed that the accuracy of the instructor's assessment is poor [22]. Therefore, the use of feedback devices in CPR practice is considered to be an important factor for learning CPR skills. Additionally, the amount of change from the post-test to the follow-up test for many parameters was marginal compared to the change from the pre-test to the post-test, and the acquired skill levels were generally retained even after 3 months. Previous studies investigating the duration of the training effect for CPR have revealed that CPR skills declined from 3 months to 12 months after training [23,24]. Spooner et al. [25] compared conventional training by an instructor (conventional group) with training using a feedback device (feedback group), and they found that the performance of the feedback group was significantly better than the conventional group for up to 6 weeks following the training. However, even in the feedback group, the retention of skills after 6 weeks was poor; the percentage of correct chest compressions immediately after training was 58.0%, but that percentage decreased to 43.1% after 6 weeks. Although direct comparisons cannot be made as the content of the feedback, the content of the practice, and the amount of practice varies, our study showed a high performance in the follow-up tests despite setting a longer follow-up period than that in the study conducted by Spooner et al. One of the factors regarding the fact that the retention of CPR skills from post-test to follow-up tests in our study were better than reports from previous studies might be the effect of overlearning [26,27]. In the second half of the practice session of our research, each parameter approached near the upper limit, and it is considered that overlearning occurred at this time. In the Spooner et al. study [25], on the other hand, they considered that overlearning had not occurred, because the score had not reached the upper limit even immediately after training. Although the impact of overlearning on retention of CPR skills is unknown and further investigations are needed, training using a feedback device can rapidly improve the learner's skills and overlearning may occur.

Moreover, the results of the present study showed that the timing and frequency of feedback affects the performance in practice sessions; concurrent, frequent feedback were superior to terminal or low frequently feedback. However, no superiority of concurrent or frequent feedback was observed for the post-tests or follow-up tests. In this study, feedback timing and frequency did not affect the retention of CPR skills; we considered that this could be due to the participants of our study having previously received CPR training. It is possible that, as a result of a previous training, the participants of this study had a high initial skill level. Sigrist et al. states that the appropriate timing and frequency for providing visual feedback depends on the relationship between the learner's skill level and the task complexity [18]. For instance, if the learner's skill level is high relative to the task complexity, excessive feedback (i.e., concurrent or frequent feedback) during practice decreases the performance in the post-test where feedback is not provided, because the learner becomes dependent on feedback [28,29]. Conversely, if the learner's skill level is low relative to the task complexity, excessive feedback during practice works effectively to increase acquisition of skills [30]. Because concurrent or frequent feedback might make complex motor tasks easier to understand by guiding the learner to correct specific movements; in contrast, an inadequate guiding effect from terminal or low frequency feedback is likely to delay the acquisition of skills [31]. All participants in the present study had already received CPR training before initiation of this study, so it is possible that the advantages (guiding effect) and disadvantages (depending effects) of concurrent or frequent feedback may have been offset. However, for those who are taking the BLS course for the first time, concurrent or frequent feedback may be effective. In contrast, when a person with a higher skill level, such as medical staff, retrains, the terminal or infrequent feedback may be effective. Further investigations are needed to reveal whether the way to give feedback to improve CPR skills efficiently depends on the skill level of the participants.

This study was subject to several limitations. First, we could not perform an a priori sample size calculation in this study. One of the reasons is that this study is the first study to examine the influence of feedback timing and feedback frequency on the acquisition of CPR skills, so there was no previous study that could be referenced for parameter inputs. The other reason was that we set a 3-month follow-up period for health sciences undergraduates, so a pilot study to estimate sample size could not be conducted. In the future, it will be necessary to conduct high-quality research with a priori sample size estimations based on the data obtained this study. Secondly, the follow-up period was short, and the influence of how the feedback was provided during the practice sessions on the decline in skill from the post-test to follow-up tests could not be detected. In this study, the follow-up period was set for 3 months when the skill starts to decline as determined by the previous research. However, it was possible to retain skills for a longer period of time by using the feedback device and probably because of the influence of overlearning; thus, it was considered that a large decline in skill was not observed except in some parameters during the 3-month follow up period. Finally, the participants of this study were limited to undergraduate students in the department of health sciences and they were already trained in CPR. Therefore, the participants of this study potentially had a high initial skill level and a high intrinsic motivation for CPR skills. Since it has been reported that skill level and intrinsic motivation are major factors affecting motor learning [17, 32], these factors may have affected the results of this study.

## Conclusions

Our study confirmed that the use of feedback devices is an important factor for implementing higher-quality CPR training, as participants’ CPR skills were retained for up to 3 months after training. Furthermore, the present study reveals that the timing and frequency of feedback strongly affect the performance during training, but only slightly affect the retention of CPR skills of health sciences undergraduate students.

## Supporting information

S1 Table Results of measuring overall score, chest compression related indices, and ventilation related indexes of each feedback group using QCPR. (XLSX)

S2 Table Results of the three-way ANOVA (F value, p value, and partial eta squared). (XLSX)
